# An Unusual Cause of Shoulder Pain: A Case Report and Review of the Literature

**DOI:** 10.7759/cureus.70488

**Published:** 2024-09-30

**Authors:** Kirti Mohan Marya, Suhani Iqbal, Archisha Marya

**Affiliations:** 1 Orthopaedic Surgery, Life Healthcare Group, Dubai, ARE; 2 Orthopaedics and Rehabilitation, Life Healthcare Group, Dubai, ARE; 3 School of Medicine, University of Liverpool, Liverpool, GBR

**Keywords:** axillary cord, axillary web syndrome, lymphatic massage, physical therapy, shoulder pain

## Abstract

Axillary web syndrome (AWS) is a rare condition characterized by the presence of a cord-like structure in the axillary region. This case report describes a 24-year-old female patient who presented with complaint of left shoulder pain and restricted range of motion with no apparent cause. She was diagnosed clinically with idiopathic AWS (IAWS) and her condition completely resolved within two weeks with four physiotherapy sessions. Imaging studies such as ultrasound and MRI are rarely helpful to diagnose this condition radiologically.

## Introduction

Axillary web syndrome (AWS) is an uncommon condition described as the formation of fibrous cord or webbing in the axillary region. Axillary web syndrome can occur because of any lymphatic or venous injury that is caused by interruption of the existing lymphatic network which adheres to subcutaneous tissue that causes cord formation [1]. It is also referred to as cording lymphedema. AWS can classically develop after surgical biopsy, removal of axillary lymph nodes or sentinel lymph node biopsy for breast cancer and after intense physical activity. This case report highlights the occurrence of idiopathic AWS (IAWS) that presented without any significant causative mechanism. The pathophysiology is yet unknown and the appropriate treatment is yet undetermined [2].

## Case presentation

**Figure 1:**
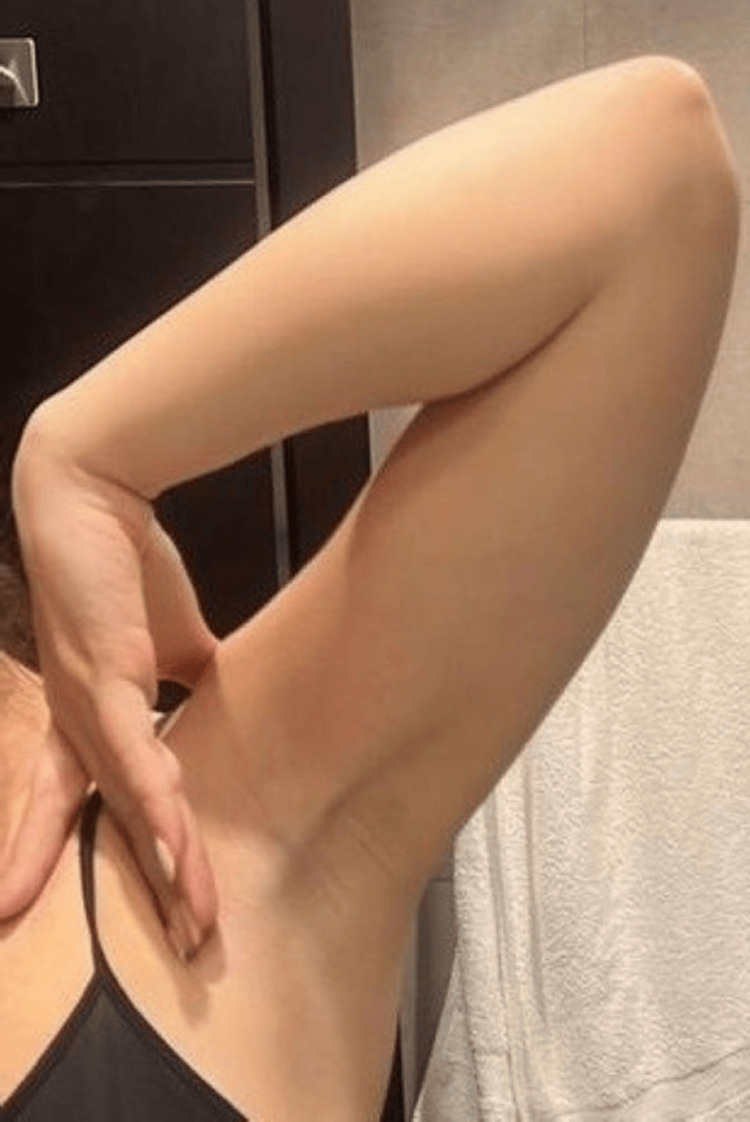
Axiallary cord in shoulder abduction

**Figure 2:**
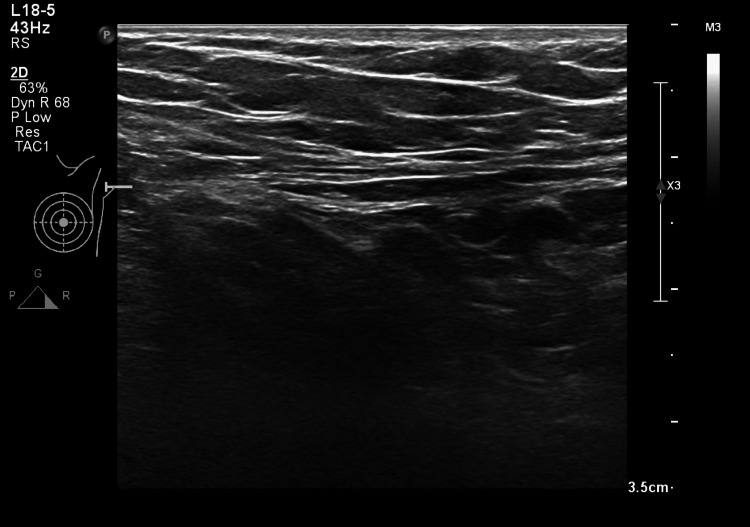
Ultrasound image of the axilla which shows no pathology.

A 24-year-old female with no significant past medical/surgical history and no history of trauma, presented with complaints of sudden-onset left shoulder pain and stiffness since five days prior to presentation to our out-patient clinic. She reported pain and discomfort in her left shoulder with limitation in overhead activity. On assessment, a palpable, tender fibrous cord-like structure was identified in the left axilla (Figure 1). The cord extended 6cm from the left axillary fold to the upper arm, with a notable restriction in shoulder abduction and external rotation. Ultrasound did not reveal any significant findings (Figure 2). Blood markers to exclude infection as well as thrombotic event, including D-dimer test, complete blood count and C-reactive protein, were normal (Table 1). Based on clinical presentation, a diagnosis of IAWS was established. 

**Table 1:** Blood Parameters RBC: red blood cells, HCT: hematocrit, MCV: mean corpuscular volume, MCH: mean corpuscular hemoglobin, MCHC: mean corpuscular hemoglobin concentration, RDW: red blood cell distribution width, RDW-CV: RDW coefficient of variation, RDW-SD: RDW standard deviation

Parameters	Patient values	Reference range
C-REACTIVE PROTEIN	0.3 mg/L	<5.0 mg/L
D-DIMER	184 ng/mL	<255 ng/mL
COMPLETE BLOOD COUNT		
RBC Count	4.28 10^12/L	3.80-4.80 10^12/L
Hemoglobin	13.1 gm/dL	12 – 15 gm/dL
HCT	39%	36-46%
MCV	91.1 fL	83-101 fL
MCH	30.5 pg	27 – 32 pg
MCHC	33.6 g/dL	31.5-34.5 g/dL
RDW-CV	0.124 %	11.0 - 16.0 %
RDW-SD	40.4 fL	35.0 - 56.0 fL
Platelet Count	303 10^9/L	150 - 410 10^9/L
White Blood Cells	9.89 10^9/L	4.00-10.00 10^9/L
DIFFERENTIAL COUNT:		
Neutrophils	62.70%	40 - 80 %
Lymphocytes	31.00%	20 - 40 %
Monocytes	5.20%	2.0 - 10.0 %
Eosinophils	1.00%	1.0 - 6.0 %
Basophils	0.1 %	<1-2 %
ABSOLUTE COUNT:		
Neutrophils	6.20 10^9/L	2.0 - 7.0 10^9/L
Lymphocytes	3.07 H 10^9/L	1.0-3.0 10^9/L
Monocytes	0.51 10^9/L	0.2- 1.0 10^9/L
Eosinophils	0.10 10^9/L	0.02 - 0.50 10^9/L
Basophils	0.01 10^9/L	0.00 - 0.10 10^9/L

**Figure 3:**
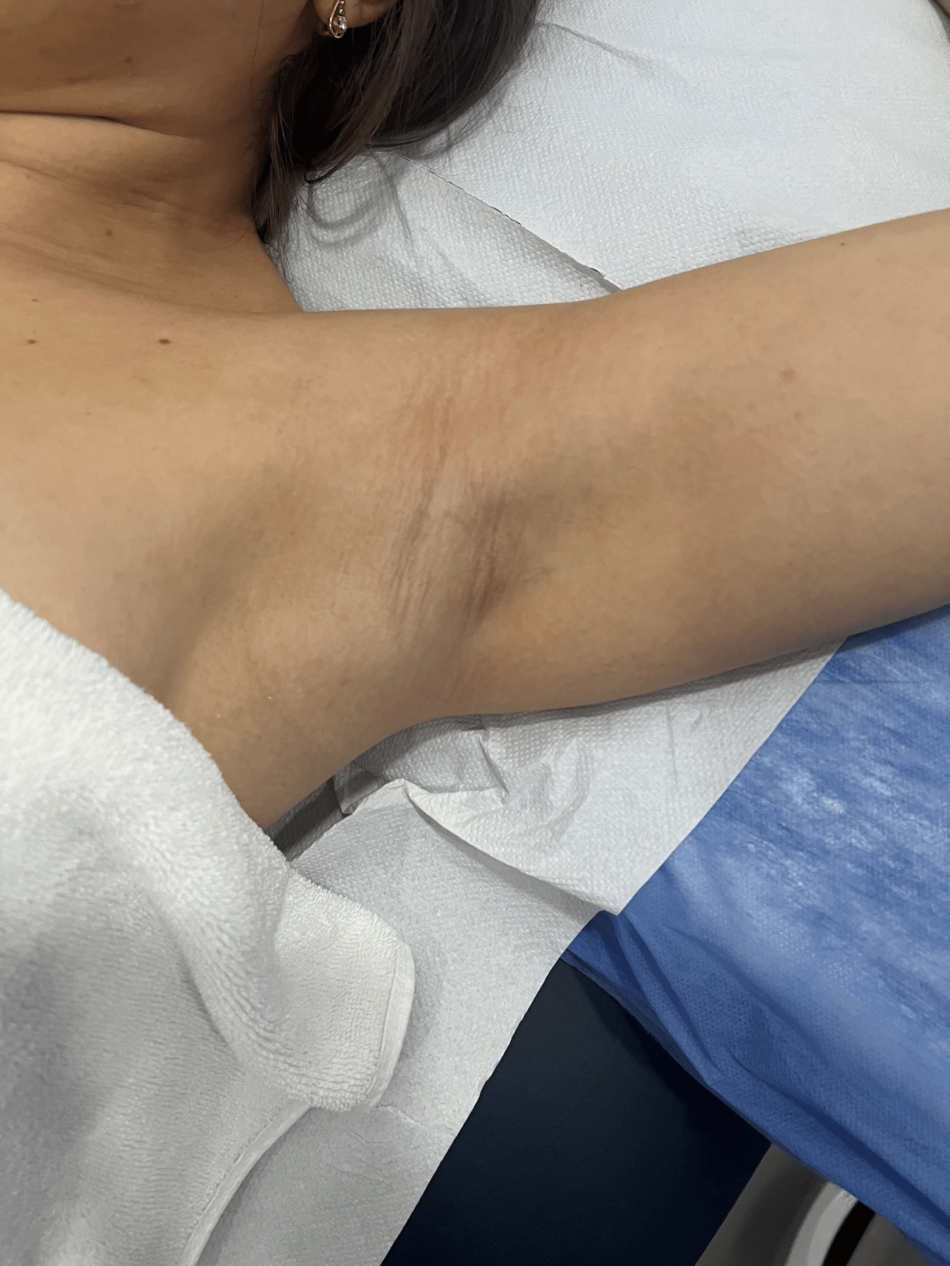
After four sessions of physiotherapy

The patient underwent physical therapy that focused mainly on soft tissue mobilization techniques/ myofascial release to break down the fibrous bands along with lymphatic drainage and in an effort to improve range of motion (ROM) stretching exercises were added. The patient felt significant difference in four physiotherapy sessions conducted over two weeks and the cord disappeared (Figure 3). No recurrence of the cord formation was observed during the follow-up period of two months. The patient’s shoulder pain disappeared and ROM was regained.

Physiotherapy thus included four sessions of treatment (40 minutes/session) and with the following protocol: a) Soft tissue massage-proximal to distal direction, b) Lymphatic massage distal to proximal, c) Ultrasonic therapy at the site, d) Shoulder range of motion exercises, e) Stretching exercises for shoulder and f) Chest wall mobilization.

## Discussion

The current case report aims to increase awareness of early and swift diagnosis of IAWS that is based on clinical assessment and medical evaluation. IAWS diagnosis is purely clinical as neither the blood flow signals in that cord-like structure show visibility on Doppler ultrasound nor the conventional ultrasound reveals any axillary lymphadenopathy [3].

AWS is often believed to be a variant of Mondor disease, which is a syndrome of sclerosing of superficial vessels [4]. AWS is a rare entity but a common cause of significant morbidity after axillary lymph node dissection for breast cancer [5]. A case of AWS in a pediatric patient was reported that was caused by strenuous physical activity and heavy weight lifting [6]. Rarely, idiopathic cases have also been reported. Based on our current knowledge first case of IAWS was reported by Demir et al. in 2017 [7]. The symptoms of IAWS included shoulder pain, limitation of shoulder abduction and flexion. Daily self-massage proved to be effective. However there is paucity of evidence that that massage can actually help in recovery or that this condition is self-limiting. Some studies speculate that physiotherapy might speed up the healing of IAWS. The treatment focuses on efficacy of lymphatic massage in reducing and eliminating cording and improving mobility. A two-person technique is recommended for cording while performing lymphatic drainage massage [8]. Table 2 reviews the reports regarding the role of physiotherapy rehabilitation [6,7,9-11] in IAWS.

**Table 2:** Summary of previously reported cases of idiopathic axillary web syndrome

Case	Year	Age	Sex	Origin	Rehabilitation	Improvement time
Demir et al. [7]	2017	40	M	Idiopathic	Yes	1 month
Puentes et al. [9]	2020	67	F	Idiopathic	No	4 month
Siddiqui et al. [10]	2023	73	F	Idiopathic	yes	1 week
İsa Cüce et al. [11]	2023	27	F	Idiopathic	yes	4 weeks
Denton et al. [6]	2024	15	F	Idiopathic	yes	3 months

## Conclusions

IAWS may be an often overlooked condition because of inability to diagnose it radiologically. This case echoes the importance of prompt diagnosis of IAWS clinically in a patient with absence of any precipitating event. Early diagnosis and multidisciplinary management can lead to quick recovery. The treatment primarily involves physical therapy aimed at improving lymphatic drainage and supporting joint mobility and reducing symptoms. One should maintain a strong index of suspicion whenever a patient presents with a short history of shoulder pain and stiffness and shows the presence of a cord-like structure in the axilla.
